# Dissipation, Processing Factors and Dietary Exposure Assessment of Myclobutanil in Tomato

**DOI:** 10.3390/molecules28165978

**Published:** 2023-08-09

**Authors:** Yanli Qi, Junli Cao, Chunyong Li, Pengcheng Ren, Shu Qin, Jindong Li

**Affiliations:** Shanxi Center for Testing of Functional Agro-Products, Shanxi Agricultural University, No. 79, Longcheng Street, Taiyuan 030031, China; qiyanli0412@126.com (Y.Q.); caojunli@sxau.edu.cn (J.C.); lichunyong2020@126.com (C.L.); zoe1946@126.com (P.R.); qinshu55@126.com (S.Q.)

**Keywords:** myclobutanil, dissipation, processing factors, tomato, dietary exposure

## Abstract

Myclobutanil residue poses a potential threat to consumers’ health. This work aims to investigate the degradation behavior, residue levels, processing factors (PFs) and dietary risk of myclobutanil in tomato. Myclobutanil was analyzed using a modified quick, easy, cheap, effective, rugged, safe (QuEChERS) method combined with ultra-performance liquid chromatography–mass spectrometry (UPLC-MS/MS), and average recoveries ranged from 82% to 102% with relative standard deviations RSDs ≤ 9.1%. After spraying myclobutanil miscible oil under field conditions, the initial concentration of myclobutanil was 0.055 mg/kg, and its dissipation followed the first-order kinetics equation with a half-life of 2.88 days. Myclobutanil was mainly present in the tomato skin, and its concentration was about four times that in the whole tomato. The initial concentration of myclobutanil in raw tomato was 0.100 mg/kg. After washing, peeling, homogenization, simmering and canning, the residual level of myclobutanil decreased to 0.067 mg/kg, 0.023 mg/kg, 0.013 mg/kg, 0.044 mg/kg and 0.041 mg/kg, respectively. Although the procedure of simmering led to an increase in myclobutanil concentration, the PFs were all less than 1 in the whole process, showing that the processing procedure significantly decreased the residual level of myclobutanil canned tomato paste in comparison with the raw agricultural commodity. Washing, peeling, and homogenization played critical roles in reducing pesticide residues. The residues of myclobutanil during the processing of tomato pose low dietary exposure risks to consumers in China, which were acceptable. However, the acute and chronic risk quotient for children revealed that it was necessary to monitor the dietary exposure of pesticide residues for children closely.

## 1. Introduction

Tomato (*Lycopersicon esculentum*) belongs to the eggplant fruit vegetable family, which is rich in organic acids, cellulose, vitamins and carotenes, and has the functions of promoting digestion, clearing heat, detoxification, cosmetology and so on. Moreover, the medicinal value of tomato in preventing cancer and cardiovascular and cerebrovascular diseases has long been recorded [1]. According to the data provided by Food and Agriculture Organization of the United Nations (FAO), tomato production of China in 2021 was about 67,636,724.84 tonnes (https://www.fao.org/faostat/en/#compare) (accessed on 3 August 2023). Diseases and pests may be responsible for the decline in tomato yield [2]. It is reported that tomato late blight could reduce tomato production by 7% [3], and tomato yellow leaf curl could result in a yield loss of up to 100% [4]. Additionally, the insect *Tuta absoluta* has become one of the most important threats to agriculture worldwide, causing large losses in open field and greenhouse tomato crops [5]. Therefore, pesticides are frequently applied to tomatoes during planting [6]. According to the reports, tomato ranks among the top ten of the most pesticide-contaminated vegetables and fruits [7]. Authorities and regulators such as the CAC (Codex Alimentarius Commission), the EU (European Union), the USEPA (United States Environmental Protection Agency), and relevant bodies in China, Canada, Japan, and elsewhere have established maximum residue limits (MRLs) for pesticides for raw agricultural commodities. The MRLs from different authorities or regulators may be different for the same pesticide. For example, the CAC stipulates that the MRL of myclobutanil in tomato is 0.3 mg/kg; the EU stipulates the MRL of myclobutanil in tomato is 0.6 mg/kg; the USEPA and Canada have established that the MRL of myclobutanil in tomato, tomato (puree) and tomato (paste) is 0.3 mg/kg, 0.5 mg/kg and 1.0 mg/kg, respectively. Japan and China have established that the MRL of myclobutanil in tomato is 2.0 mg/kg and 1.0 mg/kg, respectively.

Myclobutanil, (RS)-2-(4-chlorophenyl)-2-(1H-1,2,4-triazol-1-ylmethyl) hexanenitrile, is a systemic and foliar fungicide, which belongs to the triazole fungicide family [8] and exists as a racemate. It inhibits ergosterol biosynthesis and has good control effect on ascomycetes and basidiomycetes [9]. Myclobutanil is used to control scab, powdery mildew, downy mildew, anthrax and other diseases of vegetables, fruits, and melon crops. However, myclobutanil is classified as a moderately toxic class II compound due to its effects on antioxidant enzyme genes and animal malondialdehyde levels. It is reported that myclobutanil persists in the environment and accumulates along the food chain, which may threaten the health of consumers and cause environmental pollution [10]. In recent years, myclobutanil has caused more public concern. There are many reports about the residues of myclobutanil in agricultural products and environmental samples, including the pretreatment method [11], the dissipation dynamic [12,13], and the level of myclobutanil residues in the actual samples [14,15].

The risk assessment of residual pesticides is usually performed on the foundation of raw agricultural commodities. The effects of processing modes on pesticide residues in agricultural products have rarely been considered. Food processing is transforming raw agricultural products into other food forms for consumption by humans or animals [16,17]. Household and industrial processing, such as washing, peeling, juicing, blanching, drying, pickling and boiling, can significantly decrease or concentrate the levels of pesticide residue, or even transform pesticides to more toxic metabolites [18], which has attracted some scholars’ discussion. Wu et al. reported that the processing of washing, juicing and cooking can effectively remove pesticide residues on the surface of kumquats, cucumbers and spinach, where the PFs ranged from 0.09 to 0.7 [19]. Saber et al. showed that the residual amount of hexythiazox on strawberry surfaces decreased from 0.261 mg/kg to 0.084 mg/kg after cleaning [20]. Bian et al. found that pesticide residue was concentrated during the drying process of rose, and the PF value was >1 [21]. Xiao et al. studied the PFs of 11 typical pesticides in honeysuckle from field to table; the PFs of these pesticides were all >1 after oven, sun, and shade drying, which ranged from 3.52 to 11.2 [22]. Therefore, the influences of different treatments on the level and fate of pesticide residues in food should be considered, as they are crucial for assessing dietary intake. Myclobutanil is widely used in the cultivation of tomato. In addition to being eaten raw, fresh tomatoes are also used in tomato paste in many countries. However, myclobutanil’s change in residue level and nature during tomato paste processing had not been reported.

The aim of our study was to evaluate the fate of myclobutanil in tomato cultivation under field conditions, investigate the behavior changes of myclobutanil during tomato processing and examine processing factors and exposure risks of myclobutanil. The study will be conducive to a more accurate risk assessment in dietary exposure of myclobutanil from tomato cultivation to processing, and will provide information on the food safety and health risk evaluation of myclobutanil.

## 2. Results and Discussion

### 2.1. Method Validation and Matrix Effect

Under optimized instrument conditions, the residual method showed reliable performances in terms of linearity, recovery, and precision. The average recoveries and RSDs of myclobutanil in three matrixes are displayed in Table 1. The average recoveries in three matrices varied from 82% to 102% with the RSDs ranging from 1.2% to 9.1%. The limits of detection (LOD) and the limits of quantification (LOQ) were 0.0003 mg/kg and 0.001 mg/kg, respectively. A good linear relationship was obtained in the range from 0.001 μg/mL to 0.1 μg/mL. The regression equation of solvent calibration curves and tomato matrix calibration curves were *y* = 21,070,552*x* + 11,173 and *y* = 11,116,881*x* + 7501, respectively, and the regression coefficients were 0.9999 and 0.9996, respectively.

The matrix effect (ion suppression or ion enhancement) results from ionization competition between co-eluting components and target compounds. It is critical to ascertain the matrix effect in developing an analytical method, especially in UPLC-MS/MS analysis with an electrospray ionization source [23,24]. In the present study, the ratio of the slope of the calibration curves was used to assess the matrix effect [25,26]. The matrix effect was −47%, suggesting strong ion suppression [27]. Thus, a matrix-matched standard calibration curve was applied for accurate quantification.

### 2.2. The Dissipation of Myclobutanil in Tomato under Field Conditions

**Figure 1:**
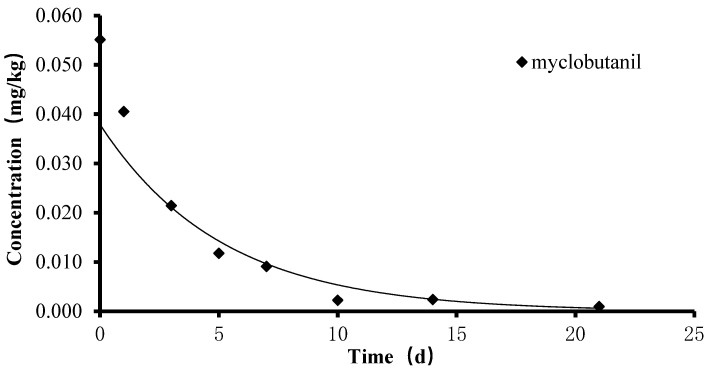
The dissipation dynamic of myclobutanil in tomato.

The magnitude of myclobutanil in tomato fruits was determined using the validated method. The dissipation curve of myclobutanil in tomato was obtained by plotting sampling time versus residual levels of myclobutanil (Figure 1). The dissipation of myclobutanil in tomato followed the first-order kinetics equation. The corresponding regression equation was *y* = 0.047e^−0.24*x*^ with a regression coefficient of 0.9347.

As shown in Figure 1, the residual concentrations of myclobutanil decreased with time. The initial deposition concentration of myclobutanil in tomato was 0.055 mg/kg following the recommended dosage, and less than the minimum MRL 0.3 mg/kg for the above organization and country. The concentration was 0.021 mg/kg after 3 days, then the concentration decreased to below the LOQ on the 21st day. The half-life of myclobutanil in tomato was 2.88 d. The results were similar to that in previous reports. Liu et al. found that the half-lives of myclobutanil in lychee were short with values ranging from 2.2 d to 3.4 d in foliar spraying mode with 1.5 times the recommended dosage. Liu et al. applied myclobutanil to wheat plant at twice the recommended dosage, and the half-lives of myclobutanil in the wheat plants ranged from 3.5 d to 4.5 d [28,29]. The residual period of myclobutanil is usually short in crops.

### 2.3. Effects of Tomato Processing on Residual Removal of Myclobutanil

The initial concentration of myclobutanil in raw tomato was 0.100 mg/kg. During canning tomato paste, five general processing procedures were investigated, and samples were collected. The residual concentrations of myclobutanil in tomato samples are listed in Table 2. In most commercial and household processing procedures, washing is the first step [30]. Washing is often the first link in the home processing process and is an important way to remove pesticide residues from raw agriculture commodities [31]. In this study, tomato fruit samples were rinsed under running water for 1 min. After washing, the residual level of myclobutanil in the whole tomato was decreased by 33% and the concentration was 0.067 mg/kg. This might relate to the physico-chemical properties of myclobutanil, such as its octanol-water partition coefficient, solubility in water, etc. According to the report of JMPR [8], the value of log Kow of myclobutanil is 2.56, and its solubility in water ranges from 115 to 132 g/L at different pH values (at 20 °C). So, washing could remove the myclobutanil from tomato skin. Tomato samples were peeled after washing, and the tomato skin and tomato pulp samples were collected. Myclobutanil was mainly present in the tomato skin, and its concentration was about four times that of the whole tomato. The residual concentration of myclobutanil in tomato pulp (0.023 mg/kg) further indicated that peeling was an effective approach to remove pesticide residues. Washing and peeling significantly reduced the pesticide residue in tomato. Similar findings have confirmed this point. Emilie Reiler et al. reported that the concentrations of dimethoate, malathion, and methyl-parathion in tomato decreased an average of 54% and 28% with washing and peeling, respectively; chlorpyriphos and ethyl-parathion almost disappeared by washing and entirely removed by peeling [32].

**Figure 2:**
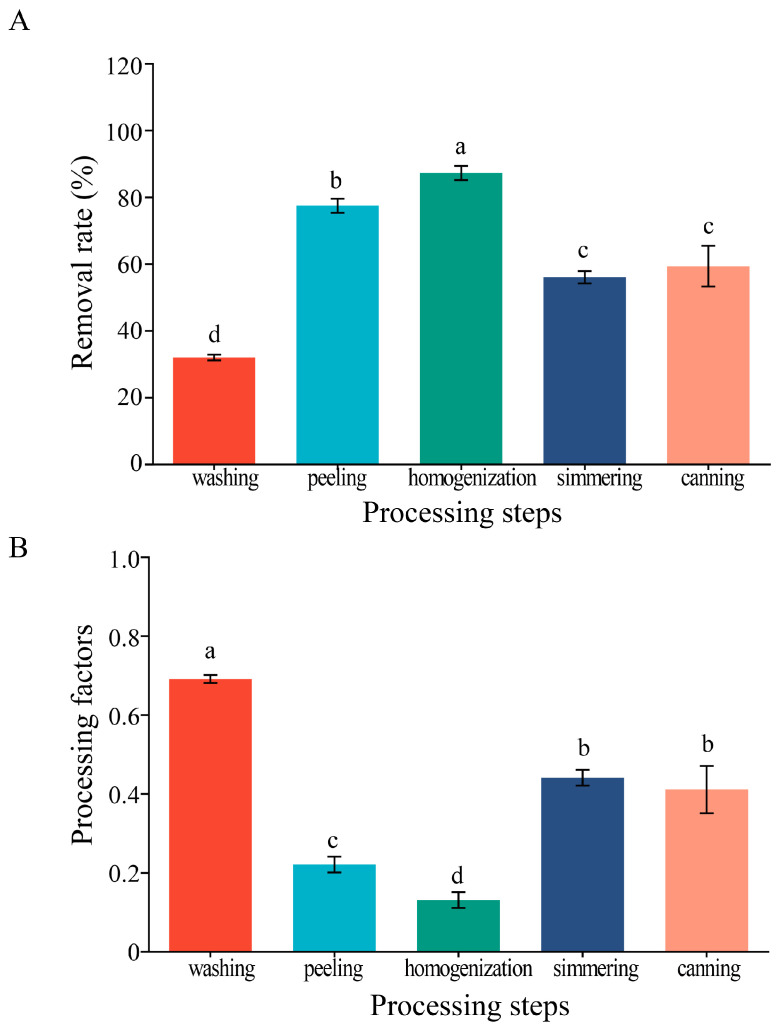
The variation of residual levels (**A**) and processing factors (**B**) of myclobutanil during the tomato processing. Abcd: different letters represent statistically significant differences among processing steps (*p* < 0.05).

After peeling, the tomato fruit was chopped into four parts. To make the sample representative, the diagonal parts were collected, then tomato juice and tomato seeds were removed, and the remaining tomato pulp was homogenized using a knife-mill Grindomix GM300 (Retsch, Haan, Germany). As shown in Figure 2A, after homogenization, the myclobutanil load was reduced by 87.3%. It was notable that myclobutanil was detected both in tomato juice and tomato seeds. Pesticides were not discovered in tomato juice and tomato seeds in previous research [32]. Myclobutanil might be transported in tomato fruits via the xylem or phloem from the other parts of the tomato plant. In addition, the mode of action of myclobutanil contributed thereto. As a systemic fungicide, compared with non-systemic fungicides, myclobutanil can be transmitted into the interior of a plant and enriched in certain specific parts.

Then, the tomato puree was simmered at 80–90 °C for 30 min to remove the excess water and form a thick tomato paste. During simmering, the residual concentration of myclobutanil tended to increase. The results were similar to previous studies. Kwon et al. found that compared to concentrations in peeled tomatoes, the concentrations of oxadixyl in puree were 192%, because 45% of water was lost during preparation of the puree [33]. Chen et al. found that when 73.0% of the water in tomato puree was evaporated, the concentrations of dinotefuran and UF increased by 114.9–404.3% in the simmering link [34]. In the study, 56% of the water in tomato puree was evaporated; this may be the reason for the increased concentration of myclobutanil in tomato. However, the residual level of myclobutanil still decreased during the process.

The tomato paste was sterilized and canned at 121 °C. Compared with tomato paste, the amount of myclobutanil in canned tomato paste was reduced by 6.8%. Previous studies have reported that sterilization eliminated or reduced pesticide residues in tomato [35]. Figure 2A shows that the removal rate of myclobutanil reached 59.3% over the entire processing procedure.

**Figure 3:**
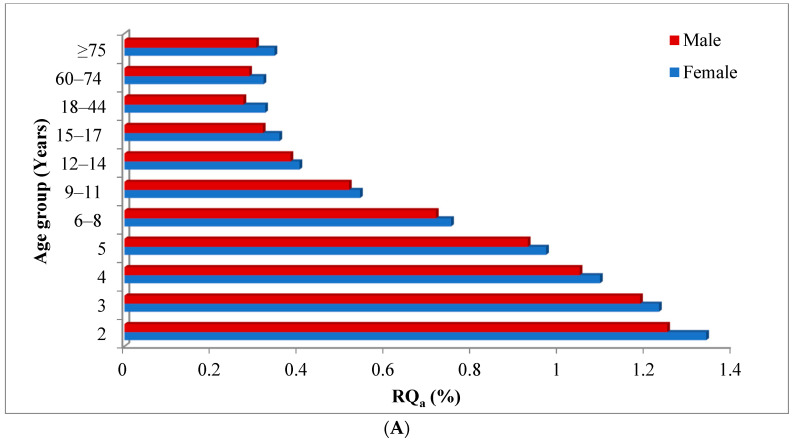

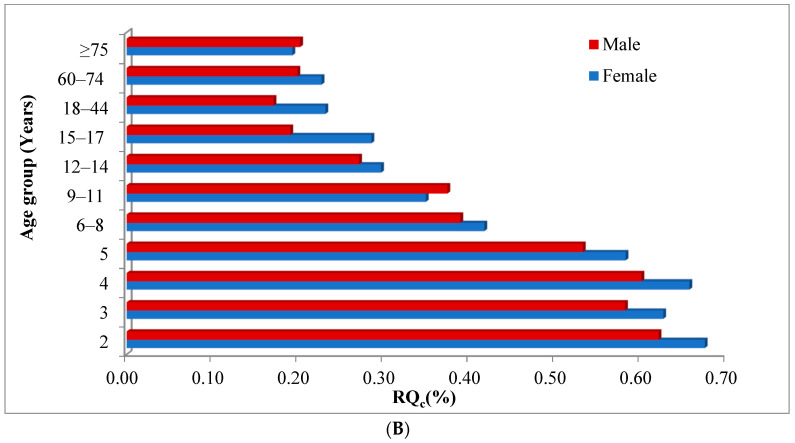
The acute risk quotient (RQa) (**A**) and the chronic risk quotient (RQc) (**B**) of myclobutanil among the tomato processing for different consumer groups.

Processing factors are important tools in risk assessment, which refine the exposure assessments for consumers regarding pesticide residues in processed food. Figure 3B illustrates the PFs of myclobutanil during processing. The PF values of washing, peeling, homogenization, simmering, and canning steps were 0.67, 0.23, 0.13, 0.44, and 0.41, respectively. After peeling and homogenization, the minimum PF was obtained, showing that these steps played an essential role in reducing pesticide residues. The increase in the processing factor indicated that water loss led to a significant enrichment in myclobutanil in the tomato samples during simmering. The PFs were all below 1, indicating that the processing decreased pesticide residues in tomato and perhaps reduced the exposure risk for consumers compared to the consumption of the raw agricultural commodity. Studies have shown that home canning procedures are safe and could reduce the residues of pesticides significantly. The PF of dinotefuran and its metabolite UF ranged from 0.081 to 0.169 among the overall tomato paste processing, which indicated the processing reducing the dinotefuran and UF levels [34].

The concentration of myclobutanil in tomato was 0.100 mg/kg, which is lower than the MRL (1 mg/kg) stipulated by China [36]. China has not set an MRL for myclobutanil in tomato paste, while the USEPA has set the MRL for myclobutanil in tomato paste as 1 mg/kg [37]. In this experiment, the concentration of myclobutanil in tomato paste was 0.041 mg/kg, which was lower than the MRL of USEPA. The final processing factor of tomato processing was 0.41, indicating that the concentration of myclobutanil was reduced during tomato paste processing. The results of this study could provide a reference for the establishment of MRL in tomato paste and the production of canned tomato paste in China.

### 2.4. Dietary Exposure Assessment of Myclobutanil in Tomato Processing

The international estimated short-term intake (IESTI) and the acute risk quotient (RQa) of myclobutanil for different age groups and genders in China are exhibited in Table 3 and Figure 3A. The values of IESTI and RQa are considered with the PF of myclobutanil among the tomato processing. As shown, the values of IESTI ranged from 0.8219 to 4.0142 µg/kg bw, which were obviously lower than the acute reference dose (ARfD) (0.3 mg/kg bw) of myclobutanil. The values of RQa ranged from 0.27 to 1.34%, which were all <100%, indicating that the acute dietary exposure risk to the myclobutanil via tomato and tomato paste consumption may have been ignored.

In general, children were considered to be the most vulnerable group because they were particularly susceptible to pesticide-related hazards [38]. As shown, the RQa values were almost negatively correlated with age, the RQa values were highest for 2-year-olds children in the range of 1.25–1.34%. Children’s dietary safety should be paid more attention. In addition, the RQa values for females were higher than for males in all age groups.

In addition, it should be noted that IESTI was based on a worst-case scenario, with the highest values used for both residue levels and diet consumption, so the calculations were overestimated and the actual risk was lower.

The values of the international estimated daily intake (IEDI) and the chronic risk quotient (RQc) are considered with the PF of myclobutanil among the tomato paste processing. Tomato belongs to dark-colored vegetables, the corresponding supervised trials median residue (STMR) values of myclobutanil in tomato is 0.08 mg/kg. The IEDI and RQc of myclobutanil were calculated for different age groups and genders in China, which are exhibited in Table 3 and Figure 3B. The NEDI values (0.2759–0.4417 mg/d) were smaller than the corresponding acceptable daily intake (ADI) values (0.4260–2.0100 mg/d) in all populations. The chronic dietary risk was higher in younger children; accordingly, it is necessary to monitor the dietary exposure of pesticide residues for children closely. Female were at greater risk than males, except the 9–11 and ≥75 age groups. However, the values of RQc ranged from 0.17 to 0.68%, which were all much less than 100%, indicating that the chronic dietary exposure risk to the myclobutanil via tomato paste consumption was acceptable.

In the present work, the potential consumer risk for pesticide was practically negligent for human health, which is in accordance with other studies [39,40,41,42]. Our results are close to the study of Yang et al. [39], which demonstrated that the RQa (<0.1–0.1%) and RQc values (<0.1–0.4%) of penthiopyrad in tomato samples showed that >90% of RQa values were <0.1% and >50% of RQc values were <0.2%. The data demonstrated that tomato samples were safe for Chinese consumers after spraying with penthiopyrad.

Human beings are at the top of the food chain and are the ultimate accumulators of chemical pollutants such as pesticides, so it is necessary to conduct dietary risk assessments for pesticides. In addition, human exposure to pesticides is determined by many factors, such as crop degradation, harvest time, and food processing. Therefore, PF is a tool to evaluate the dietary risk assessment of raw agricultural commodities [43,44]. Jankowska et al. adjusted for the dietary risk using processing factors [41].

According to the above results, the residual levels of myclobutanil in tomato after processing pose low dietary exposure risks to consumers in China. The data demonstrated that tomato and tomato paste samples were safe for different Chinese consumer groups after spraying with myclobutanil.

## 3. Materials and Methods

### 3.1. Chemicals and Reagents

Myclobutanil standard material was purchased from Dr. Ehrenstorfer (Augsburg, Germany). Myclobutanil formulation was supplied by the Yifan Biotechnology group (Wenzhou, China). Methanol (LC-MS grade) was obtained from Merck KGaA (Darmstadt, Germany). Acetonitrile and formic acid (HPLC grade) were purchased from Thermo Fisher Scientific (Shanghai, China). Sodium chloride (analytical grade) purchased from Beijing Reagent Company (Beijing, China). Water was purchased from Watson’s (Guangzhou, China). WondaPak QuEChERS dispersive tubes (2 mL) were purchased from SHIMADZU-GL (Shanghai, China).

### 3.2. Field Experiments and Sampling

Field trials were conducted in Jinzhong City, Shanxi Province, China. Each plot was 15 m^2^. Two separate field experiments were performed to investigate the residual behavior of myclobutanil in tomato under field conditions and during processing. Each experiment was performed in triplicate, and a control experiment was conducted. For the dissipation fate study, myclobutanil miscible oil (10%) was sprayed one time in a single dosage as recommended. Tomato fruit samples were collected at 2 h (0 d), 1 d, 3 d, 5 d, 7 d, 10 d, 14 d, and 21 d after application. The collected tomato samples were transported to the laboratory as soon as possible and were homogenized and stored in a freezer at below −18 °C. For the tomato-processing study, the same commercial formulation was sprayed three times at double the dose as recommended and the application interval was 7 d. Two days after the last spraying, about 10 kg of tomatoes were collected for the following test.

### 3.3. Processing Treatments

**Figure 4:**
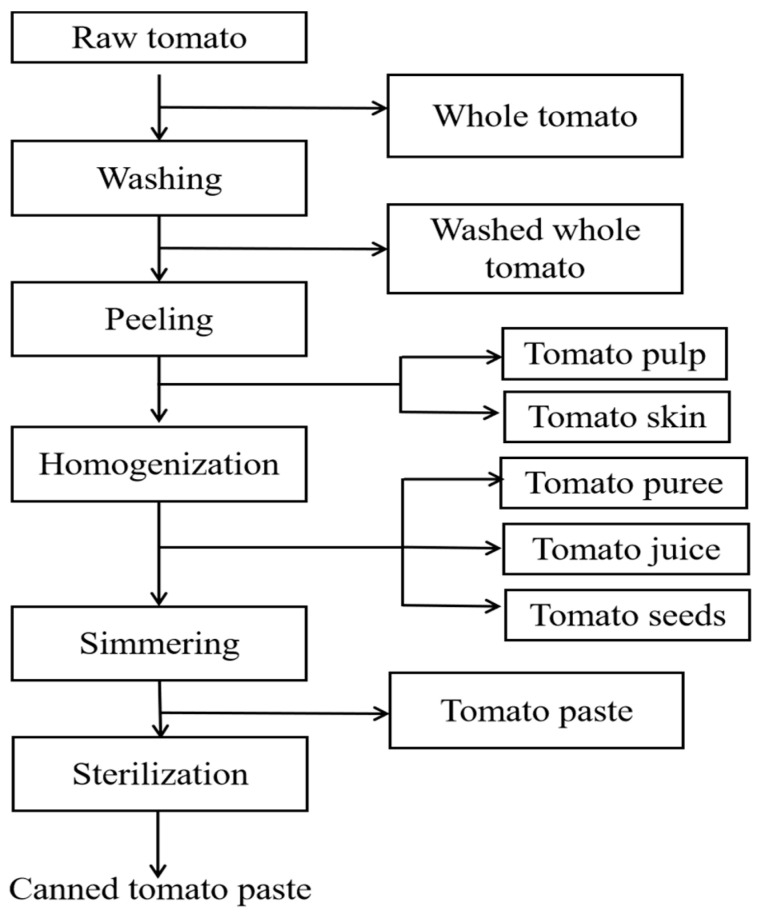
Processing of Tomato.

Part of the raw tomato samples were analyzed without processing treatment, some of the raw tomatoes were peeled, and the remainder sample was processed to tomato paste. The production procedures of canned tomato paste generally involve five steps: washing, peeling, homogenization, simmering, and canning [34]. The processing steps and sampling points are shown in Figure 4. In processing, the whole tomato, washed whole tomato, washed tomato skin, tomato pulp, the tomato puree, tomato juice, tomato seeds, tomato paste, and canned tomato paste were collected and assayed to demonstrate the variation of residue levels and processing factors as pertaining to myclobutanil.

### 3.4. Extraction and Cleaning of Samples

QuEChERS [45,46] is a kind of pretreatment technology that has emerged in recent years and has been applied rapidly. With its advantages of being quick, easy, cheap, effective, rugged and safe, it is widely used in pesticide residue detection. So, a modified QuEChERS method was used to extract and purify myclobutanil in tomato.

Representative samples of 5 g of homogenized material, 5 g of sodium chloride, and 10 mL of acetonitrile were added to a 50 mL centrifuge tube. The samples were mixed using an advanced multi-tube vortexer for 5 min at 2500 rpm, and then centrifuged for 5 min at 8000 rpm. A volume 1 mL of the supernatant was transferred to a QuEChERS dispersive tube containing 25 mg of Primary Secondary Amine (PSA) and 25 mg of C18, vortexed for 2 min at 2500 rpm, and subsequently centrifuged for 2 min at 2500 rpm. A volume of 0.5 mL of the upper layer was placed into a 2 mL tube, to which we added 0.5 mL water, mixed it, then filtered the material through 0.22 μm filters for UPLC-MS/MS analysis.

### 3.5. Instrument Analysis

UPLC-MS/MS is a powerful analytical technique that combines the separation capabilities of liquid chromatography with the high sensitivity and selective mass analysis capabilities of triple quadrupole mass spectrometry. As a chromatographic identification instrument, mass spectrometry has a fast speed, good separation and wide applications [38]. So, the residual level of myclobutanil in tomato was analyzed using UPLC-MS/MS. The instrument method was provided by Waters engineers.

Chromatographic separation analysis of myclobutanil was conducted on a Waters Acquity UPLC I―class binary solvent manager (Waters, Milford, MA, USA), which was equipped with a BEH C18 column (50 mm × 2.1 mm, 1.7 μm) (Waters, USA). The column temperature was 40 °C, the sample volume injected was 5 μL and the flow rate was 0.3 mL/min. The mobile phase consisted of phase A: water with 0.1% formic acid and 4 mM ammonium acetate, and phase B: methanol with 0.1% formic acid and 4 mM ammonium acetate. The gradient elution was 0–1 min 50% phase A, 1.1–4.0 min 5% phase A, and 4.1–5.0 min 50% phase A.

Waters Xevo TQ-S Micro MS/MS (Waters, USA) was used for mass spectrometric analysis. The ESI (electrospray ionization) source was used, which operated in positive ionization mode. MS analyses were performed using the MRM (multiple reaction monitoring) mode. The source voltage was 3000 V and the desolvation temperature was 400 °C. The desolvation gas flow was 800 L/h and source temperature was 150 °C. The quantitative and qualitative ions of myclobutanil were *m*/*z* 288.9/69.9 and 288.9/124.9, and the collision energies were 24 V and 38 V, respectively. The cone voltage for myclobutanil was 36 V.

### 3.6. Method Validation

The analytical methods of myclobutanil in tomato samples were verified using the accuracy, precision, linearity, and matrix effect (ME), according to SANTE/11312/2021 [47].

To evaluate the accuracy and precision of the analytical method, the standard of myclobutanil was spiked to three blank matrices (tomato, tomato juice, and tomato seeds) at 0.01 mg/kg, 0.1 mg/kg, and 0.5 mg/kg. Each experiment was replicated five times. The recovery (%) and relative standard deviation (RSD, %) were calculated. The LOD and LOQ were defined as the concentrations that produced signal-to-noise (S/N) ratios of 3 and 10, respectively.

To evaluate the linearity and ME, a series of myclobutanil working standard solutions (0.001 μg/mL, 0.005 μg/mL, 0.01 μg/mL, 0.05 μg/mL, and 0.1 μg/mL) were prepared by diluting the stock standard solution with acetonitrile, and the curves in solvent and tomato matrix were prepared, respectively.

### 3.7. Data Analysis

#### 3.7.1. Dissipation Kinetics Analysis

The dissipation kinetics of myclobutanil in tomato followed the first-order kinetics Equation (1); the half-life (*t*_1/2_) was calculated from the *k* value using the following Equation (2) [6]:

C*_t_* = C_0_e^−*kt*^ (1)

*t*_1/2_ = ln2/*k* (2)

C*_t_* and C_0_ represent the residual level of myclobutanil (mg/kg) at time *t* and the initial concentration of myclobutanil (mg/kg), respectively. *k* is the dissipation rate constant.

#### 3.7.2. PF Calculation

According to the agricultural industry standard of China (NY/T 3095―2017): Guideline for the testing of pesticide residues in processed agricultural commodities, the PF is equal to residues in processed agricultural commodities (mg/kg) divided by residues in raw agricultural commodities (mg/kg). When the PF is greater than 1, it indicates a concentration increase in processed agricultural commodities during processing, whereas a decrease in the residue concentration is expressed by a factor of less than 1 [48,49].

The removal rate was calculated as follows:

Removal rate (%) = (C_0_ − C*_p_*)/C_0_ × 100 (3)

where C_0_ is the initial concentration (mg/kg), and C*_p_* is the concentration after processing (mg/kg).

#### 3.7.3. Acute (Short-Term) Intake Risk Assessment

The IESTI and the RQa was calculated using the method recommended by Joint Meeting on Pesticide Residues (JMPR) [50].

(4) $$\mathrm{IESTI}=\frac{\mathrm{Ue}\times \left(\mathrm{HR}\;\times \mathrm{PF}\right)\times v+\left(\mathrm{LP}-\mathrm{Ue}\right)\times \left(\mathrm{HR}\;\times \mathrm{PF}\right))}{\mathrm{bw}}$$

(5) $$\mathrm{RQa}=\frac{\mathrm{IESTI}}{\mathrm{AR}fD}\;\;$$

Ue is edible portion of the unit weight (kg); the Ue of tomato is 0.175 kg from FAO/WHO GIFT platform; HR is the highest residue in tomato sample of the edible portion found in data from supervised trials data, from which the MRL was derived (mg/kg), the HR of myclobutanil in tomato is 0.38 mg/kg from China; LP is the highest large portion provided (97.5th percentile of eaters), in kg of food per day; bw is the average body weight for a population age group (kg); and ARfD is the acute reference dose; the value of myclobutanil is 0.3 mg/kg bw, based off a JMPR report. When RQa ≤ 100%, indicating acute dietary exposure risk is acceptable; otherwise, it is not acceptable.

#### 3.7.4. Chronic (Long-Term) Intake Risk Assessment

The IEDI and the RQc was calculated using the method recommended by the WHO [51].

(6) $$\mathrm{IEDI}=\frac{\sum \mathrm{STMRi}\times \mathrm{PF}\times \mathrm{Fi}}{\mathrm{bw}}$$

(7) $$\mathrm{RQc}=\frac{\mathrm{IEDI}}{\mathrm{ADI}}$$

STMRi is the supervised trials median residue, in the composite sample of the edible portion found in data from supervised trial data, from which the MRL was derived (mg/kg); the STMR of myclobutanil in tomato is 0.08 mg/kg from China; the STMRs of myclobutanil in other registered crops are the MRLs; Fi is the dietary consumption (kg/d), with be in kilograms; ADI is the acceptable daily intake, and the value of myclobutanil is 0.03 mg/kg bw from China. When RQa ≤ 100%, it indicates that the acute dietary exposure risk is acceptable; otherwise, it is not acceptable.

#### 3.7.5. Statistical Analysis

The data were evaluated statistically using one-way ANOVA analysis with SPSS 25.0 software. Fisher’s Least Significant Difference (LSD) test was used to determine the differences among means if significant differences were found. Differences with *p* < 0.05 were considered statistically significant.

## 4. Conclusions

In the study, a viable residue analysis approach was established and successfully used to identify the residual magnitude of myclobutanil in tomato. The variations of the residual level of myclobutanil in tomato in the field and during processing were investigated. The residual levels were less than the MRL of China. Washing, peeling, and homogenization were essential in reducing pesticide residues. The PFs ranged from 0.13 to 0.67. Compared to the raw agriculture commodity, with processing, the residual level of myclobutanil in canned tomato paste was significantly reduced. The dietary intake risk results verified that the tomato and tomato paste samples were safe for consumers in China when myclobutanil was sprayed in accordance with the recommended approach. This work provides valuable guidance on the establishment of MRL for myclobutanil in tomato paste and the production of canned tomato paste in China.

## Figures and Tables

**Table 1:** Average recoveries and RSD of myclobutanil in tomato, tomato juice, and tomato seeds (*n* = 5).

Matrix	Spiked Levels (mg/kg)	Average Recovery (%)	RSD (%)
Tomato	0.01	96	1.5
	0.1	92	1.9
	0.5	93	2.1
Tomato juice	0.01	97	1.8
	0.1	96	1.8
	0.5	94	2.5
Tomato seed	0.01	102	9.1
	0.1	94	1.2
	0.5	82	6.7

**Table 2:** The residual concentrations of myclobutanil in the tomato samples.

Sample	Concentration ± SD (mg/kg)
Raw tomato	0.100 ± 0.009
Washed whole tomato	0.067 ± 0.024
Tomato skin	0.395 ± 0.052
Tomato pulp	0.023 ± 0.001
Tomato puree	0.013 ± 0.001
Tomato juice	0.009 ± 0.001
Tomato seeds	0.027 ± 0.009
Tomato paste	0.044 ± 0.003
Canned tomato paste	0.041 ± 0.006

**Table 3:** The dietary risk assessment of myclobutanil among tomato processing for different consumer groups.

Age Group	Gender	BW (kg)	Consumers P97.5 (g/d)	IESTI μg/(kg bw d)	RQ_a_%	Dietary Consumption (kg)					IEDI mg/d	Acceptable Daily Intake mg/d	RQc %
						Flour and Its Products	Other Grains	Light Vegetables	Citrus	Dark Vegetables			
2 years	Female	14.2	15.8654	4.0142	1.34	0.0577	0.0079	0.0353	0.0449	0.0381	0.2877	0.4260	0.68
	Male	14.8	7.3099	3.6930	1.23	0.0589	0.0093	0.0309	0.0491	0.0340	0.2759	0.4440	0.62
3 years	Female	15.3	12.6582	3.2850	1.10	0.0577	0.0079	0.0353	0.0449	0.0381	0.2877	0.4590	0.63
	Male	15.8	11.2613	2.9125	0.97	0.0589	0.0093	0.0309	0.0491	0.0340	0.2759	0.4740	0.58
4 years	Female	17.2	12.6582	2.2563	0.75	0.0769	0.0079	0.0429	0.0715	0.0372	0.3392	0.5160	0.66
	Male	17.9	11.2613	1.6277	0.54	0.0821	0.0092	0.0458	0.0755	0.0322	0.3229	0.5370	0.60
5 years	Female	19.4	12.6582	1.2092	0.40	0.0769	0.0079	0.0429	0.0715	0.0372	0.3392	0.5820	0.58
	Male	20.2	11.2613	1.0704	0.36	0.0821	0.0092	0.0458	0.0755	0.0322	0.3229	0.6060	0.53
6–8 years	Female	24.6	6.2500	0.9722	0.32	0.0906	0.0103	0.0880	0.0477	0.0404	0.3082	0.7380	0.42
	Male	25.8	6.2101	0.9607	0.32	0.1041	0.0116	0.0955	0.0509	0.0375	0.3016	0.7740	0.39
9–11 years	Female	34.1	6.2500	1.0367	0.35	0.1034	0.0125	0.1072	0.0545	0.0472	0.3574	1.0230	0.35
	Male	35.8	6.2101	3.7614	1.25	0.1218	0.0126	0.1140	0.0577	0.0545	0.4022	1.0740	0.37
12–14 years	Female	45.9	6.2500	3.5623	1.19	0.1285	0.0115	0.1201	0.0645	0.0530	0.4092	1.3770	0.30
	Male	48.4	6.2101	3.1444	1.05	0.1438	0.0141	0.1258	0.0652	0.0494	0.3942	1.4520	0.27
15–17 years	Female	51.5	3.8251	2.7864	0.93	0.1317	0.0117	0.1312	0.0749	0.0552	0.4417	1.5450	0.29
	Male	57.6	3.4671	2.1511	0.72	0.1910	0.0168	0.1443	0.0700	0.0337	0.3302	1.7280	0.19
18–44 years	Female	56.7	3.8251	1.5502	0.52	0.1268	0.0129	0.1592	0.0821	0.0430	0.3948	1.7010	0.23
	Male	67.0	3.4671	1.1466	0.38	0.1569	0.0123	0.1750	0.0845	0.0314	0.3447	2.0100	0.17
60–74 years	Female	57.3	3.3093	0.9561	0.32	0.1168	0.0196	0.1621	0.0810	0.0429	0.3912	1.7190	0.23
	Male	63.9	3.2401	0.8219	0.27	0.1373	0.0193	0.1778	0.0862	0.0386	0.3823	1.9170	0.20
≥75 years	Female	53.0	2.6508	0.8613	0.29	0.0905	0.0152	0.1294	0.0718	0.0305	0.3078	1.5900	0.19
	Male	60.5	3.3273	0.9099	0.30	0.1126	0.1590	0.1574	0.0843	0.0364	0.3678	1.8150	0.20

## Data Availability

The data presented in the study are available from the authors upon request.

## Sample Availability

Samples of the compounds are available from the authors upon reasonable request.
